# Significantly Increased Public Interest in Major Depressive Disorder During the COVID-19 Pandemic: Insights From a Google Trends Analysis

**DOI:** 10.7759/cureus.21228

**Published:** 2022-01-14

**Authors:** Chencheng Li, Qinyi Tan, Manxing Zou, Liang Zeng, Muyun Kang, Lisha Chen

**Affiliations:** 1 Center for Studies of Education and Psychology of Ethnic Minorities in Southwest China, Southwest University, Chongqing, CHN; 2 Department of Natural Medicinal Chemistry, China Pharmaceutical University, Nanjing, CHN; 3 Office of Medical Education Research, Chongqing Medical University, Chongqing, CHN; 4 Academic Affairs Office, Southwest Medical University, Luzhou, CHN; 5 Traditional Chinese Medicine, Affiliated Traditional Chinese Medicine Hospital of Southwest Medical University, Luzhou, CHN

**Keywords:** mental illness, psychology health, pandemic, google trends, major depressive disorder (mdd), covid-19

## Abstract

Background

As early as before the coronavirus disease 2019 (COVID-19) pandemic, nearly one billion people worldwide suffered from mental health problems. Of all the mental health conditions, major depressive disorder (MDD) is the leading cause of global health-related burden. During the COVID-19 pandemic, many uncertain factors affecting mental health accumulated, such as virus transmission, blockade and ban, public transport restrictions, closure of schools and enterprises, and reduction of social interaction, which led to an increase in the potential risk of MDD, further increasing the global health-related burden.

Methodology

To better clarify the public interest in major depressive disorder during the COVID-19 pandemic, a Google Trends analysis was employed with data from December 2019 to December 2021, taking the cumulative diagnosis rate and cumulative mortality rate of COVID-19 as the reference standard, The changes in public interest and behavior in online searching for major depressive disorder in the three countries most affected by the severe acute respiratory syndrome coronavirus 2 (SARS-CoV-2) virus (i.e. the United States, Brazil, and India) were evaluated.

Results

We observed that during the COVID-19 pandemic, public interest in major depressive disorder increased significantly on the Internet. At the same time, compared with the United States, this upward trend is more prominent in India and Brazil. The study found that the major depressive disorder search index of the United States reached the maximum at the end of September 2021, the major depressive disorder search index of Brazil reached the maximum at the beginning of July 2021, and the major depressive disorder search index of India reached the maximum at the beginning of June 2021. The above time nodes are the first turning point of decline after the continuous surge of COVID-19 confirmed cases in the United States, Brazil, and India, indicating that there is an important time correlation between the surge of COVID-19 cases and the public online search term major depressive disorder.

Conclusion

The Google Trends analysis shows that public interest in major depressive disorder is on the rise under the COVID-19 pandemic and that COVID-19 may be associated with MDD. These findings deserve further exploration, especially as a growing body of research reports suggests that the COVID-19 pandemic has led to a surge in the prevalence of MDD. The epidemic alerts the vast majority of countries to urgently strengthen mental health systems and provide patients with the necessary interventions based on the determinants of poor mental health.

## Introduction

The Global Burden of Diseases, Injuries, and Risk Factors Study (GBD) 2019 shows that major depressive disorder (MDD) is one of the most disabling mental disorders, ranking among the top 25 main causes of global health-related burden [1]. During the coronavirus disease 2019 (COVID-19) pandemic, the global prevalence of major negative disorder (MDD) has increased by nearly one-fourth (1/4th). Mental health problems have become another public crisis encountered by many countries after the invasion of the severe acute respiratory syndrome coronavirus 2 (SARS-CoV-2) virus. A new mental disorder study from Austria pointed out that the symptom changes of MDD are largely affected by seasons, especially winter [2]. More importantly, the COVID-19 pandemic began in the winter of 2019, which means that is the peak season for MDD patients. Therefore, there may be an overlap between the seasonal surge of psychiatric symptoms after non-SARS-CoV-2 virus infection and psychiatric symptoms after SARS-CoV-2 virus infection. In order to better clarify the public interest in major depressive order during the COVID-19 pandemic, a Google Trends analysis was employed to evaluate the changes in public interest in online searching for major depressive order information in the three countries most affected by the SARS-CoV-2 virus (i.e., the United States, Brazil, and India).

## Materials and methods

Study design, period, and location

The U.S., Brazilian, and Indian public who searched online over the Internet for major depressive disorder (MDD) between December 1, 2019, and December 1, 2021, were included in this Google Trends analysis. According to the global COVID-19 case data shown on Google Maps, the United States, Brazil, and India are the top three countries in terms of cumulative COVID-19 diagnosis and mortality rates, and their societies and publics are seriously affected in all aspects. Assessing the link between the public health-related burden in the United States, Brazil, and India with the COVID-19 pandemic better reflects the reliability of Google Trends analytics in disease prediction, prevention, and control than in other countries, and provides constructive solutions for mental health systems in the vast majority of countries and regions around the world.

Data retrieval and processing

Data were reported in relative (Internet) search volumes (RSVs) with the search term “major depressive disorder” and were compared among the United States, Brazil, and India. Google RSVs are indicative of percentages relative to the peak search volume observed during a particular time period and scaled by the total search volume for each specific search term. The exact numbers do not mean the absolute search volume, but instead, the data are normalized and reported in the study on a scale from 0 to 100.

Statistical analysis

Based on Google RSVs data from the United States, Brazil, and India, Microsoft Excel (Microsoft Corporation, Redmond, WA) was used to make linear predictions of public interest in online searches for major depressive disorder (MDD) during the COVID-19 pandemic to assess the correlation between COVID-19 and MDD.

## Results

**Figure 1:**
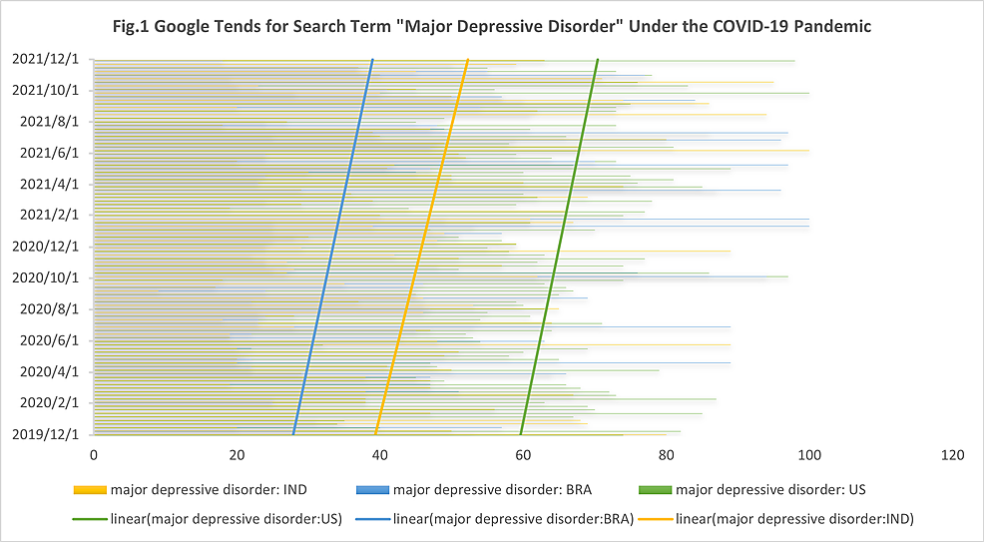
Google Trends for search term major depressive disorder during the COVID-19 pandemic

Based on Figure 1, there was a significant increase in public interest in online searches for major depressive disorder information during the COVID-19 pandemic. At the same time, this upward trend is more pronounced in India and Brazil than in the United States. Our analysis points out that the major depressive disorder search index reached its maximum in the United States at the end of September 2021, the major depressive disorder search index in Brazil reached its maximum in early July 2021, and the major depressive disorder search index reached its maximum in early June 2021 in India. The above time nodes are the first inflection points in the decline of confirmed COVID-19 cases after the continuous surge in the United States, Brazil, and India, which indicates a significate temporal correlation between the surge in COVID-19 cases and the online search for major depressive disorder.

## Discussion

This is the first time to utilize a Google Trends analysis to clarify public interest in major depressive disorder during the COVID-19 pandemic, and it shows that public interest in major depressive disorder has grown surprisingly in the context of the COVID-19 pandemic. At the same time, compared with the United States, the growth trend is more pronounced in Brazil and India, which implies an important correlation between the surge in the prevalence of MDD and the COVID-19 pandemic.

During the COVID-19 pandemic, many uncertain factors affecting mental health have accumulated. The spread of the virus, blockade bans, public transportation restrictions, school and business closures, and reduced social interactions have led to a significantly increased risk of MDD. The more severely affected countries and regions by the COVID-19 pandemic, the higher the prevalence of MDD [3]. In fact, long before the COVID-19 pandemic, major depressive disorder (MDD) was one of the main causes of global health-related burden, and most research reports pointed out that the mental health systems of most countries around the world are under-resourced, making it difficult to provide effective interventions for patients with MDD [4]. Especially in low-income and middle-income countries, more than 75% of patients with MDD are not treated [5]. Therefore, we speculate that during the COVID-19 pandemic, the interruption of psychiatric clinics and the reallocation of medical resources for the key treatment of SARS-CoV-2 virus infections may lead to a large number of patients with underlying MDD) being rejected and ignored by the hospitals, and instead, looking for their own symptoms through the Internet [6].

The alarming growth of major depressive disorder online search trends is worrisome. This warns that most countries need to urgently strengthen their mental health systems and provide patients with necessary interventions based on the decisive factors of poor mental health. Healthcare professionals and medical media personnel are responsible for disseminating public health knowledge about the impact of COVID-19 on mental health, providing the public with clear access to medical services, and encouraging the public to seek medical treatment in time for mental disorders such as headaches, lose sleep, retardation of thought, low mood, etc. Meanwhile, it is necessary to consider the local context and vulnerable populations and prioritize key principles, such as inclusivity, stigma reduction, and human rights, in the context of implementing the UN sustainable development goals (SDGs) [7]. Besides, in terms of treatment and medication, especially for MDD patients should give priority to cognitive behavioral therapy (CBT), interpersonal therapy (IPT), and other therapies, and antidepressants such as tricyclic antidepressants (TCAs) and selective serotonin reuptake inhibitors (SSRIs) must be used with caution under the premise of medical advice.

There are still limitations associated with the research that need to be improved. More specifically, these searches may represent cases in Brazil and India, largely due to limited Internet access in certain regions.

In addition, this study does not include searches performed on search platforms other than Google Search. In this study, in the absence of patient-level data on granularity, i.e., without a detailed history of MDD, there were some speculative observations. However, this study can serve as a preliminary source for a broad population perspective of MDD in the context of the COVID-19 pandemic [8].

Further study to respond to the following questions is supported:

During the COVID-19 pandemic, the surge in public interest in online search for major depressive disorder is only driven by temporary anxiety, or is there always under-reporting of the burden of real cases of MDD?

Has the COVID-19 pandemic hindered the public from going to medical institutions in time for medical treatment and, instead, being encouraged to look for their illnesses online?

Compared with other countries, why is India’s most significant interest in MDD information during the COVID-19 pandemic? Compared with MDD patients in other countries, is the genetic structure of MDD patients in India a predisposing factor for mental illness?

Under the COVID-19 pandemic, does the high attention of the public to MDD imply that the symptoms of real cases of MDD are continuing to worsen?

In general, under the COVID-19 pandemic, the surge of public interest in major depressive disorder has aroused vigilance. This requires the vast majority of countries and regions around the world to take immediate measures, encouraging the public to seek medical advice in a timely manner and providing regular follow-up services for MDD patients.

## Conclusions

In conclusion, the Google Trends analysis pointed out that the public interest in online searches for major depressive disorder during the COVID-19 pandemic has grown tremendously. Compared with the United States, the growth trend is more prominent in Brazil and India. This shows that there is an important relationship between COVID-19 and MDD. We speculate that the COVID-19 pandemic may be one of the main reasons for the recent surge in MDD cases. How to alleviate this increasingly serious global health-related burden is currently a major challenge faced by most countries and regions. Definitely, it is a significant chance to rebuild their mental health systems. Google Trends is considered an effective and convenient online tool for the real-time regional monitoring of the COVID-19 pandemic, as well as the related interest of web users in MDD, which could indicate the potential risk of MDD and help identify MDD patients.
